# The Becker Method: A Straightforward Method for Accurate Dental Age Assessment in the Clinical Setting

**DOI:** 10.1155/ijod/9488570

**Published:** 2025-08-07

**Authors:** Maya Dora Davidovitch, Avi Leibovich, Stella Chaushu

**Affiliations:** ^1^Faculty of Dental Medicine, Hebrew University of Jerusalem, Jerusalem, Israel; ^2^Department of Orthodontics, Faculty of Dental Medicine, Hebrew University of Jerusalem, Hadassah Medical Center, Jerusalem, Israel

**Keywords:** Becker method, chronological age, Demirjian method, dental age, dental maturation

## Abstract

**Background:** Accurate dental age (DA) assessment is important in the clinical decision-making process of pediatric dentists and orthodontists. While clinical methods based on tooth emergence are inaccurate, techniques using tooth calcification for assessing DA are more precise but impractical for clinical use due to their complexity. Becker proposed a more straightforward and practical method for accurate DA assessment based on root apex closure. However, its level of accuracy has not been determined.

**Aim:** To evaluate the reliability of the Becker method by comparing it to the Demirjian method.

**Design:** This cross-sectional study analyzed panoramic radiographs from a cohort of 377 Israeli children and adolescents aged between 8 and 15 years. DA was evaluated using the Demirjian and Becker methods and compared with the chronological age (CA) of each participant.

**Results:** Both methods overestimated DA when compared to CA, with the Becker method showing a closer agreement with CA. The average overestimation of Becker method was 0.659 years for females and 0.123 years for males, and of Demirjian method 1.167 and 0.713 years, respectively.

**Conclusions:** The Becker method can provide clinicians with a user-friendly, hands-on diagnostic tool, ensuring ease of application in clinical settings without compromising on precision or reproducibility.

**Summary**

• Evaluating dental age (DA) holds significant importance in the clinical decision-making process for both orthodontists and pedodontists.
• DA assessment based on teeth eruption is often inaccurate and misleading. More accurate methods based on tooth formation are too complex for clinical implementation.
• The Becker method is a practical approach for assessing DA in clinical settings, based on tooth formation rather than eruption, making it more accurate.

## 1. Introduction

Methods to determine the dental age (DA) of children and adolescents are of great value, especially in pediatric dentistry and orthodontics [1, 2]. Before initiating treatment, clinicians assess the extent of dental development by determining the patient's DA. The evaluation of DA is crucial in effectively planning orthodontic treatment. It aids in assessing the timing of tooth eruption, and identifying teeth that are impacted [3]. DA assessment helps in determining the optimal timing for orthodontic treatment, including the selection of fixed or removable appliances and planning for orthodontic tooth extractions. Assessing DA is also important for determining the most appropriate timing and options for treatment in pediatric dentistry. This includes decisions regarding the extraction of over-retained primary teeth, space maintenance strategies, and the optimal timing for the extraction of severely compromised permanent first molars to facilitate spontaneous space closure [4]. Furthermore, DA assessment is essential in forensic science for determining chronological age (CA), as there exists a strong correlation between CA and DA [5–8].

Assessment of DA can be determined radiographically or by direct clinical visualization of the dentition. The latter method is based on the emergence of teeth in the oral cavity, as observed clinically without the need for auxiliary diagnostic tools. This method has validity because it has been shown that the average timing of the eruption of teeth is fairly constant and can be employed in ascertaining an age range for a given individual. However, it would be a mistake to depend solely on the timing of tooth eruption as a method for estimation. While different groups of teeth typically erupt at specific times (with half to two-thirds of the final root length) [9], this process can be affected by local and environmental factors such as habits, available space in the dental arch, extraction or early loss of primary teeth, and tipping or impaction of teeth, eventually leading to significant variations in eruption timing [3, 10, 11].

In contrast to tooth emergence, tooth calcification is a continuous process that is assessed by permanent records such as dental radiographs and is unaffected by the local conditions mentioned above. In addition, the stages of calcification of the permanent dentition during dental development are predominantly governed by genetic factors rather than environmental influences [12–17]. Therefore, assessment of dental calcification has been showed to be a more accurate method of determining DA than tooth emergence.

Several radiographic methods to estimate DA have been proposed. These methods define the stages of mineralization of each individual tooth as observed in dental radiographs, and describe them in words and in illustrated diagrams and tables [18]. Empirical definitions of these stages were displayed in the classic works of Schour and Massler [19], Moorrees et al. [14, 20], Nolla [13], Demirjian et al. [21], Koyoumdjisky-Kaye et al. [22], and other notable sources [3].

### 1.1. The Demirjian Method

The most frequently used and accepted analysis of DA assessment is the Demirjian's method [21]. Originally described in 1973 by Demirjian, Goldstein, and Tanner, this method is based on a large French-Canadian sample with DA estimation being determined by the development of the permanent dentition of a subject's mandibular left quadrant of permanent teeth, excluding the third molars. Tooth formation is ranked according to eight stages (A–H). The determined developmental stage for each tooth is assigned a biologically weighted score. The data is converted to a DA by using available standard tables for each gender. The cumbersome nature of the Demirjian method has made it unsuitable for practical use in a clinical setting, thus making it more appropriate as a tool for clinical research.

### 1.2. Becker's Method

Becker proposed a simpler method of DA assessment in 1997 and 1998 [3, 23, 24] with the express purpose of enabling DA diagnosis quickly, at the orthodontist's chairside. The method is considerably simpler, as it does not require tables or calculations. Instead, it diagnoses directly from a qualitative examination of the panoramic radiograph or full-mouth periapical survey. The Becker method requires the observer to be familiar with the average CAs for the permanent teeth's emergence in a specific population. This method differs from other methods by its focus on the identifiable completion of formation and closure of the root apices of the teeth, which normally occur approximately 3 years after the tooth has achieved functional eruption [25]. The disappearance of the root-forming dental papilla which signifies the closure of the root apex is the most accurate feature to recognize, identify and diagnose; therefore, it can be used as a reliable baseline from which to begin the evaluation of the patient's DA [3, 23, 24]. The process follows a stepwise approach where the teeth are categorized into groups according to their eruption timing (Table 1). The DA is determined based on the last group of teeth that exhibit closed apices. This is most reliably determined when a subject has reached at least the DA of 9 years, because prior to this, the apices of all unerupted and erupting permanent teeth are still open.

**Figure 1:**
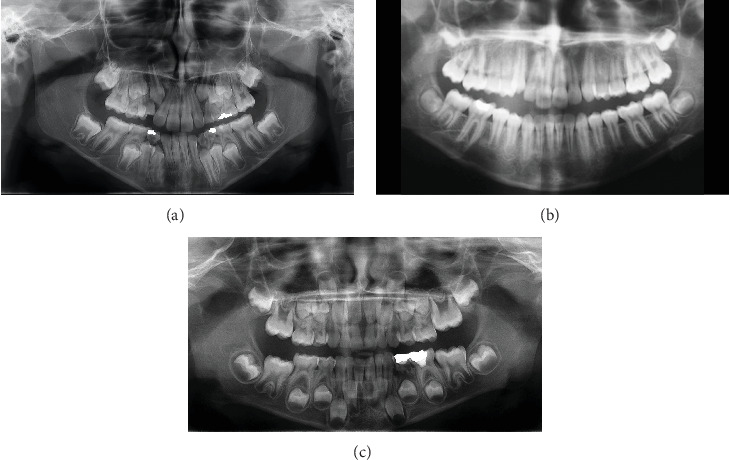
(A) Panoramic radiograph showing dental age of 9.5 years. (B) Panoramic radiograph showing dental age of 10.5–11 years. (C) The panoramic radiograph displays all teeth with open apices.

Thus, in Figure 1A the DA is 9.5 as the permanent molars and mandibular lateral incisors are the final set of teeth to exhibit closed apices. Likewise, in older individuals (Figure 1B), where the maxillary central and lateral incisors are the final set of teeth with closed apices, the estimated DA ranges from 10.5 to 11.

Prior to reaching the DA of 9 years (Figure 1C), none of the permanent teeth will have apexified roots. Thus, the observer need to estimate percentages of root development, crown formation and, in much younger children, even the initiation of crown calcification. Assessing age in this manner is more subjective and less accurate. In Figure 1C the root formation in the lower central incisors and 1st permanent molars/lateral incisors is approximately 2/3 to 1/2 respectively indicating a DA of around 6 years.

The primary goal of this study is to compare the Becker method, which is based on the timing of root apex closure, to the Demirjian method in determining DA in relation to CA, in order to evaluate the reliability of the former.

## 2. Materials and Methods

### 2.1. Subjects

Panoramic radiographs of 377 subjects who presented for treatment in the Department of Orthodontics postgraduate clinic at the Hebrew University Faculty of Dental Medicine were utilized to determine DA in this cross-sectional investigation. Inclusion criteria for subjects in this study were: 8–15 years of age, with high-quality radiographic diagnostic records. Subjects were excluded if they had a history of dental trauma, congenitally missing teeth, or syndromes, or if they had undergone any previous orthodontic treatment. The sex and age distribution of the study sample are shown in Table 2. The study received ethical approval from the Helsinki Committee of Hadassah University Hospital (Approval No. 0129-22).

Each subject's CA at the time of being x-rayed was calculated.

DA was estimated according to the methods described by Demirjian et al. [21] and Becker [3]. These evaluations were conducted by the main examiner (author MDD), who was blinded to the CA values. Initially, the examiner assessed the DA using one technique and then, several weeks later, used the second method to analyze the data.

### 2.2. Statistical Analysis

Differences between DA and CA were analyzed using the Wilcoxon signed rank test for paired samples with a non-normal distribution. A *p*-value of <0.05 was considered statistically significant. Simple linear regression analysis and correlation (Pearson's *r* coefficient) was used to study the association between DA and CA for girls and boys separately, as well as for the whole group. These analyses were performed using Prism version 10.2.2, GraphPad Software, Boston, Massachusetts, USA.

### 2.3. Intra and Interobserver Reliability

A randomly selected 25 male and female samples each (*n* = 50) were first assessed twice during an interval of at least 2 weeks, with repeat evaluations by another examiner (AL). Both examiners were blinded to the CA values. To evaluate reproducibility and accuracy, both interobserver and intraobserver assessments were performed using intraclass correlation coefficient (ICC). The calculation was performed using the intraclass_corr() function from the pingouin library In Python [26].

## 3. Results

Reliability of the measurements within the same examiner (Intraexaminer—Table 3) was calculated using ICC [26] and showed high values (0.944–0.966) for both examiners using both Demirjian and Becker methods. Consistency between examiners (Interexaminer—Table 4) was also calculated using ICC [26] and were high for both the Demirijian and Becker methods with coefficient scores ranging from 0.918 to 0.971.

Comparison between Demirjian's and Becker's DA estimation methods were analyzed separately for females and males, as well as collectively for both genders across the different age groups. The results are displayed in Tables 5 and 6, for females and males, respectively. The mean CA of the study group was 12.104 (SD = 1.938) years for females, 12.056 (SD = 2.042) years for males, and 12.08 (SD = 1.989) years for the entire study group.

**Figure 2:**
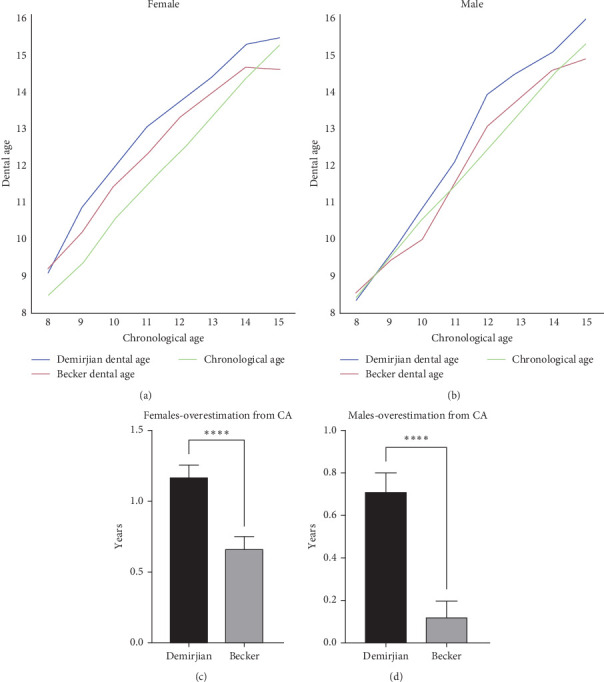
Comparison between DA calculation: Becker vs. Demirjian, in females and males—Graphical comparison of the calculated DA for females (A and C) and males (B and D) using the Becker and Demirjian methods with their CA, highlighting the differences in age estimation techniques. *p*-Values are shown (*⁣*^*∗∗∗∗*^<0.0001).

As shown in Figure 2, and Tables 5 and 6, both the Demirjian and Becker methods show an overall overestimation of the DA compared to the CA, for both females and males, with the Demirjian method exhibiting a significantly greater overestimation (Figure 2C,D) compared to the Becker method. It was also found that several exceptions were seen, namely, that in the 9, 10, 15 male and 15 female age group (Tables 5 and 6), the Becker methods did not exhibit an overestimation of CA while the Demirjian method did.

**Figure 3:**
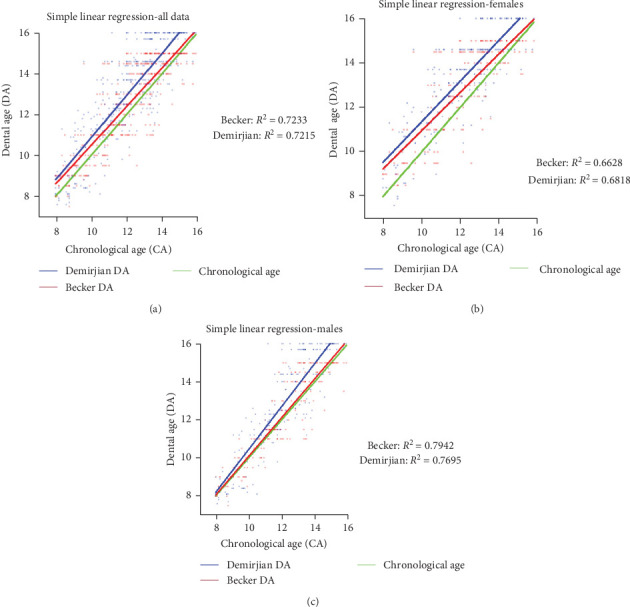
Linear regression of chronological age (CA) and dental age (DA): The linear regression relationship between the estimated DA using the Demirjian and Becker methods and the CA is examined for all patients (A), females (B), and males (C), along with the corresponding *R*-squared values for each method.

Simple linear regression was used to evaluate the relationship between the two DA calculation methods and CA (Figure 3). Upon examining the graph representing all patients irrespective of gender, both the Demirjian and Becker methods exhibit similar goodness-of-fit values (Figure 3A; *R*^2^ = 0.72). Slightly lower values are observed when analyzing the graphs based on gender, with higher values observed in the males group (Figure 3B,C). However, both methods still show similar values. The slopes of the graphs are quite similar and closely resemble the CA values. In the graph representing all patients, the slopes for the Demirjian and Becker methods were 1.03 and 0.946, respectively, and were not significantly different (*p*=0.07). In the female group, these values were 0.9102 and 0.8587 (*p*=0.02), respectively, and in the male group, the values were 1.129 and 1.019 (*p*=0.01), respectively. When compared to the slope of CA (value = 1), both methods exhibit a good level of fitness to the CA graph.

**Figure 4:**
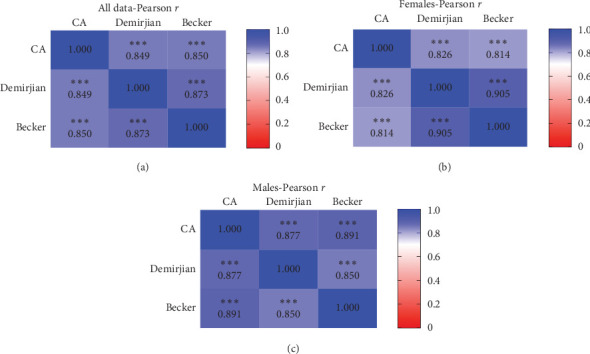
Correlation of chronological age (CA) and dental age (DA) estimates—Pearson's correlation coefficient for the combined study sample (A), females (B), and males (C), showing the strength of the correlation between the CA and the age estimated using both study methods (the Demirjian and Becker methods). The color bar-darker colors indicate higher correlation. *p* Values are shown (*⁣*^*∗∗∗*^<0.001).

To better assess the correlation between the DA calculation methods and the CA, we conducted a Pearson *r* correlation test (Figure 4). In the graph representing all patients irrespective of gender, there was a strong, positive, and significant correlation between the DA methods and the CA, with correlation coefficients of 0.849 (*p* < 0.0001) for Demirjian and 0.85 (*p* < 0.0001) for Becker (Figure 4A). Similarly, in the female group, the correlation coefficients were 0.826 (*p* < 0.0001) and 0.814 (*p* < 0.0001), respectively (Figure 4B). The male group exhibited higher values, with correlation coefficients of 0.877 (*p* < 0.0001) and 0.891 (*p* < 0.0001), respectively (Figure 4C). We observed similar coefficients values when directly comparing the Demirjian method to the Becker method, ranging from 0.85 to 0.905 (*p* < 0.0001) depending on the specific group analyzed (Figure 4).

## 4. Discussion

Accurate assessment of DA is essential for both dental and forensic applications. Evaluation of the accuracy of DA estimation techniques commonly involves a comparison between the estimated DA and the actual CA [7, 27–29]. Besides accuracy, the DA estimation technique should be simple, reproducible, and easily adaptable to different populations.

Both the Demirjian method and the Becker method exhibited high ICC values for DA assessment (Tables 3 and 4), indicating consistent results between observers. Consistent outcomes were also observed when the same observer conducted measurements at different times.

Given that the examiners in our study had varying levels of experience, the remarkable reliability we observed among them implies that extensive experience is unnecessary for effectively using either method.

Numerous studies have already showed a significantly high correlation between DA assessment and CA [8]. In our study, we show similar results using the Demirjian and Becker methods for both genders, with correlation coefficients reaching up to 0.9.

Our results also revealed a consistent trend of overestimating DA compared to CA when applying the Demirjian method, consistent with previous reports [30–32]. Notably, Demirjian et al. [21] acknowledged the potential limitation of their standards, derived from a sizable sample of mid-20^th^-century French-Canadian origin, and suggested caution when applying the method to different populations, advocating for potential adjustments. Existing literature indicates the necessity of population-specific calibration for DA assessment methods, given the variability in dental development across diverse populations and also over time due to secular trends [33].

Comparison of the Becker to the Demirjian methods in this study revealed that both tend to overestimate DA compared to CA (Figure 3), however, the Becker method demonstrated a significantly smaller mean difference from CA in both genders (The average overestimation of Becker method was 0.659 years for females and 0.123 years for males, and of Demirjian method 1.167 and 0.713 years, respectively, Tables 5 and 6; Figure 2C,D).

In forensic medicine, the generally accepted margin of error between estimated DA and CA for individuals ranges from 0.5 to 1.0 years [7]. While comparable data are not available in orthodontics and pedodontics, it is reasonable to assume a similar range, as greater discrepancies could significantly influence clinical decision-making processes that rely on DA assessments. Our study showed that for both males and females of all ages, the Becker method estimated DA within an acceptable range of CA. However, the Demirjian method showed greater discrepancies in several age groups (Tables 5 and 6). Therefore, the Becker method appears more accurate for estimating DA in the Israeli population.

Esan et al.'s [34] meta-analysis of studies comparing the Willems and Demirjian methods showed similar results. Willems, an improved method using a modern European reference population and revised maturity scores, addresses the Demirjian method's overestimation of DA. Their findings indicated Willems provided a better fit to CA across diverse populations, with average overestimations of 0.29 years (females) and 0.26 years (males), compared to Demirjian's 0.72 and 0.62 years, respectively [34].

Similarly, the Becker method's superiority likely stems from using contemporary Israeli eruption data [22] and its methodological simplicity as the Becker method focuses on the easily identifiable and most reliable feature—root apex closure. This biological landmark is less subjective than the complex staging system used in the Demirjian method, potentially reducing assessment variability.

The Becker method's simplicity lies not only in its ability to calculate DA effortlessly but also in its independence from fixed tables and figures, unlike other methods. Adapting the Demirjian method to any population is cumbersome, requiring changes to all tables and illustrations; however, the Becker method's flexibility allows easy adaptation to various populations by simply updating eruption timing data within the stepwise assessment, based on available population-specific data [33]. Such updates, however, require validation through additional research.

We also discovered additional trends in different age groups. One notable finding was that the Becker method underestimated DA at the age of 15. This discrepancy could be attributed to the Demirjian method's extension beyond the age of 15, a range surpassing the limit of the Becker method, reaching up to the age of 16 (Tables 5 and 6, and Figures 2 and 3).

The limitations of this study include potential sample population bias, as the research was confined to Israeli children seeking orthodontic treatment who had panoramic radiographs available. This may restrict the generalizability of the findings to other ethnic groups. Additionally, in children below the age of nine, where the Becker method's predictive accuracy might be weaker since none of the teeth display closed apices, making assessment is more challenging (Figure 1C; Tables 5 and 6). Although our data did not show a decrease in accuracy of the Becker method for ages below nine, it is important to note that this age group was under-represented since it is typically less prevalent in orthodontic populations due to their dental developmental stage and presents unique challenges for age estimation methodologies. The Becker method should therefore be used cautiously with younger children.

## 5. Conclusion

Both the Becker and Demirjian methods demonstrated a consistent but significant overestimation of DA up to age 15, with the Becker method showing the closer agreement with CA. The findings presented herein suggest that the Becker method may be considered as a comparatively simple and reliable alternative, encouraging further exploration of its application in diverse clinical settings.

## Figures and Tables

**Table 1:** Stepwise assessment procedure of the Becker method.

1	The first stage is to study the panoramic radiograph, to locate the mandibular permanent central incisor teeth and check for apexification. The discovery of an open apex/incomplete root formation or of an apical papilla renders the diagnosis as less than 9 years of age and disqualifies further analysis. A positive finding of apexification determines a DA of 9 years. This observation warrants that the same determination now be performed on the next tooth that is normally scheduled to erupt, that is the first permanent molars
2	If confirmation of full apical root closure of the permanent molars is made, the patient's DA estimation is confirmed and adjusted to 9–9.5 years, and the next normally erupting tooth is examined in the same fashion
3	In parallel to the first permanent molars, the mandibular permanent lateral incisors are examined. If these are found to have undergone apexification then the patient's estimated age is advanced to 9.5 years
4	The maxillary central incisors are next in line and closed apices would indicate a DA of 10 years
5	The next tooth expected to erupt is the maxillary lateral incisor—eruption time 7½−8 years, apex closure—10½−11 years. However, since the rate of development of these teeth is known to be variable, it is advised to exclude them from the diagnostic process
6	Apexification of the mandibular canines and first premolars is then examined with their apexification corresponding to a DA of 12–13 years
7	Apexification of the maxillary first premolars (DA of 13–14 years)
8	As with the maxillary lateral incisors, the mandibular second premolars have also been shown to display highly variable development, therefore, their assessment should also be foregone
9	If closed apices of the maxillary canines, this would raise the DA to 14–15 years
10	The final stage of DA is determined from the second permanent molars (15 years)

*Note:* The evaluated teeth are grouped according to eruption timing and checked for roots apices closure. The dental age is established according to the last group showing closed apices.

**Table 2:** Distribution of the study sample according to age and sex.

Age	Males	Females	Total
8	17	14	31
9	22	15	37
10	18	26	44
11	32	23	55
12	29	33	62
13	38	40	78
14	22	21	43
15	15	12	27
Total	193	184	377

*Note:* The study sample is divided into eight age groups and according to gender.

**Table 3:** Intra-rater reliability in DA estimation.

Intra-rater scores	Observer 1	Observer 2
Becker	0.944	0.956
Demirjian	0.97	0.966

*Note:* ICC scores for the Becker and Demirjian methods.

**Table 4:** Inter-rater reliability of dental age assessment methods.

Inter-rater scores	1	2
Becker	0.918	0.967
Demirjian	0.971	0.954

*Note:* ICC scores for the Becker and Demirjian DA assessment methods, as measured by two different raters. (1) ICC scores of the first evaluation in each method made by observers 1 and 2. (2) ICC scores of the second evaluation in each method made by observers 1 and 2.

**Table 5:** Comparison of DA estimation methods in females.

Female									
Age (*n*)	Mean CA (SD)	Mean Becker (SD)	Mean Demirjian (SD)	^a^Age difference Becker – CA	^a^Age difference Demirjian – CA	^b^Age difference Demirjian – Becker	*p*-Value Becker	*p*-Value Demirjian	*p*-Value Demirjian vs. Becker
8 (14)	8.476 (0.3)	9.21 (0.699)	9.093 (1.062)	0.738	0.617	−0.121	0.002	0.091	0.441

9 (15)	9.311 (0.255)	10.167 (1.41)	10.847 (1.563)	0.856	1.536	0.68	0.015	0.002	0.012

10 (26)	10.545 (0.272)	11.48 (1.62)	11.962 (1.509)	0.936	1.417	0.481	0.01	<0.001	0.038

11 (23)	11.511 (0.27)	12.33 (1.02)	13.083 (0.821)	0.815	1.572	0.757	0.001	<0.001	0.008

12 (33)	12.389 (0.31)	13.333 (1.467)	13.776 (1.217)	0.944	1.387	0.442	0.001	<0.001	0.002

13 (40)	13.373 (0.26)	14.025 (1.11)	14.45 (1.205)	0.652	1.08	0.427	0.001	<0.001	0.002

14 (21)	14.413 (0.28)	14.69 (0.4)	15.305 (0.87)	0.278	0.892	0.614	0.004	0.000	0.002

15 (12)	15.292 (0.27)	14.625 (0.569)	15.492 (0.804)	−0.667	0.2	0.867	0.002	0.569	<0.001

Total (184)	12.104 (1.94)	12.764 (2.044)	13.271 (2.136)	0.66	1.167	0.518	<0.001	<0.001	<0.001

*Note:* Evaluating the estimation of dental age in females using the Demirjian and Becker methods compared to CA, with statistical analyses, including SD and *p*-values for method comparison.

Abbreviations: CA, chronological age; DA, dental age; SD, standard deviation.

^a^Dental age minus chronological age.

^b^Demirjian DA estimation minus Backer DA estimation.

**Table 6:** Comparison of DA estimation methods in males.

Male									
Age (*n*)	Mean CA (SD)	Mean Becker (SD)	Mean Demirjian (SD)	^a^Age difference Becker – CA	^a^Age difference Demirjian – CA	^b^Age difference Demirjian – Becker	*p*-Value Becker	*p*-Value Demirjian	*p*-Value Demirjian vs. Becker
8 (17)	8.43 (0.34)	8.55 (0.52)	8.347 (0.53)	0.116	−0.084	−0.2	0.329	0.517	0.03

9 (22)	9.49 (0.3)	9.4 (0.618)	9.54 (1.185)	−0.089	0.048	0.14	0.566	1.000	0.986

10 (18)	10.53 (0.27)	10 (1.54)	10.84 (1.185)	−0.532	0.306	0.84	0.30	0.246	0.038

11 (32)	11.44 (0.304)	11.55 (0.85)	12.116 (1.187)	0.104	0.673	0.569	0.803	0.001	0.018

12 (29)	12.43 (0.33)	13.086 (1.233)	13.95 (1.26)	0.652	1.514	0.862	0.004	0	0.031

13 (38)	13.443 (0.262)	13.82 (1.302)	14.58 (1.5)	0.373	1.136	0.763	0.057	0.000	0.011

14 (22)	14.462 (0.29)	14.59 (0.59)	15.09 (0.91)	0.129	0.629	0.5	0.074	0.007	0.001

15 (15)	15.29 (0.29)	14.9 (0.387)	15.97 (0.09)	−0.394	0.672	1.07	0.000	0.000	<0.001

Total (193)	12.056 (2.042)	12.179 (2.335)	12.77 (2.629)	0.123	0.713	0.567	0.0164	0	<0.001

*Note:* Evaluating the estimation of dental age in males using the Demirjian and Becker methods compared to CA, with statistical analyses, including SD and *p*-values for method comparison.

Abbreviations: CA, chronological age; DA, dental age; SD, standard deviation.

^a^Dental age minus chronological age.

^b^Demirjian DA estimation minus Backer DA estimation.

## Data Availability

The datasets used and/or analyzed during this study are available from the corresponding author upon reasonable request.
